# Geographical Gradients in Argentinean Terrestrial Mammal Species Richness and Their Environmental Correlates

**DOI:** 10.1100/2012/819328

**Published:** 2012-09-17

**Authors:** Ana L. Márquez, Raimundo Real, Marta S. Kin, José Carlos Guerrero, Betina Galván, A. Márcia Barbosa, Jesús Olivero, L. Javier Palomo, J. Mario Vargas, Enrique Justo

**Affiliations:** ^1^Biogeography, Diversity, and Conservation Research Team, Department of Animal Biology, Faculty of Sciences, University of Malaga, 29071 Malaga, Spain; ^2^Departamento de Ciencias Naturales, Facultad de Ciencias, Universidad Nacional de La Pampa, Avenida Uruguay 151, Santa Rosa 6300, Argentina; ^3^Instituto de Ecología y Ciencias Ambientales (IECA), Facultad de Ciencias, Universidad de la República, Iguá 4225, esq. Mataojo, Montevideo 11400, Uruguay; ^4^“Rui Nabeiro” Biodiversity Chair, CIBIO, University of Évora, 7000-890 Évora, Portugal; ^5^Division of Biology, Imperial College London, Silwood Park Campus, Ascot, Berkshire SL5 7PY, UK

## Abstract

We analysed the main geographical trends of terrestrial mammal species richness (SR) in Argentina, assessing how broad-scale environmental variation (defined by climatic and topographic variables) and the spatial form of the country (defined by spatial filters based on spatial eigenvector mapping (SEVM)) influence the kinds and the numbers of mammal species along these geographical trends. We also evaluated if there are pure geographical trends not accounted for by the environmental or spatial factors. The environmental variables and spatial filters that simultaneously correlated with the geographical variables and SR were considered potential causes of the geographic trends. We performed partial correlations between SR and the geographical variables, maintaining the selected explanatory variables statistically constant, to determine if SR was fully explained by them or if a significant residual geographic pattern remained. All groups and subgroups presented a latitudinal gradient not attributable to the spatial form of the country. Most of these trends were not explained by climate. We used a variation partitioning procedure to quantify the pure geographic trend (PGT) that remained unaccounted for. The PGT was larger for latitudinal than for longitudinal gradients. This suggests that historical or purely geographical causes may also be relevant drivers of these geographical gradients in mammal diversity.

## 1. Introduction

There is a long recognized and widely documented latitudinal gradient of species richness (SR) for terrestrial, marine, and freshwater taxa, with a general increase towards the equator [1–8]. This gradient has been maintained for at least 270 Myr [9, 10] and is the oldest and most fundamental pattern regarding life on earth [11–13]. However, the mechanisms responsible for this biodiversity gradient remain poorly understood despite great efforts by researchers in recent centuries [14], although many hypotheses have been proposed to explain it [15, 16]. Understanding the mechanisms underlying the latitudinal biodiversity gradient may be one of the most important challenges for biologists in the 21st century, given the alarming rates of biodiversity loss as a result of human activities [13, 17, 18]. Originally, a general mechanism was sought (based on climate) that would explain the latitudinal gradient in SR, but no consensus was reached and more synthetic multifactor approaches have appeared in recent decades [10].

Some authors maintain that latitude *per se* does not affect SR [19–21] and consider that the correlation between latitude and SR is entirely spurious, being latitude used as a variable for historical reasons and because it can be easily and objectively measured at a global scale. However, latitude is a physical characteristic of rotating planets (note that a still sphere does not have poles or latitude) and cannot be reduced to an arbitrary coordinate axis to be used in spatial analyses. Thus, both ecology and geography may influence latitudinal patterns in SR [22].

Longitude can also play a role in shaping biogeographical patterns of SR. However, the longitudinal biodiversity gradient is not as common and well documented as the latitudinal one. For example, Korpimäki and Marti [23] detected a longitudinal gradient in the dietary diversity of North American raptors related to the regional trend in prey assemblages, where the number of mammal prey species markedly increases from east to west. Longitudinal and other spatial trends may also appear depending on the characteristics of the territory analysed. 

Environmental factors are responsible for most of these geographic trends [10]. Real et al. [24] found that environmental factors explain most of the geographic variation of mammal SR in Argentina. The present study attempts to deepen our knowledge of the geographical structure of Argentinean mammals by identifying specific geographical trends of terrestrial mammal SR in Argentina and possible environmental explanations for these trends. Nonenvironmental mechanisms (e.g., disturbance or history), although of importance, are not addressed because they are outside the scope of this work. The following questions are addressed. (i) Which are the main geographical trends shown by the SR of Argentinean terrestrial mammals? (ii) How does broad-scale environmental variation influence the kinds as well as numbers of mammal species along these geographical trends? (iii) Are there pure geographical trends not accounted for by the environmental variables?

## 2. Materials and Methods

### 2.1. Study Area

Argentina is the seventh largest country in the world (surface area 2,791,810 km^2^ excluding Antarctica and the South Atlantic islands). Its longest axis is aligned from north to south, from near the Tropic of Capricorn down to Cape Horn. Argentina is included within the subtropical-temperate zone but shows unusual climatic diversity, from northern tropical climates to southern cold ones [25]. In addition, the Andean mountain range, which stretches from the north to the south of the country along its western border, exerts a major influence on climate by acting as barrier for the moist winds from the Pacific Ocean. The Andes break the rains on the western hillsides while the eastern hillsides remain very dry [26]. Furthermore, rivers tend to flow from mountainous western to flat eastern regions, contributing to the longitudinal species gradient [25].

**Figure 1:**
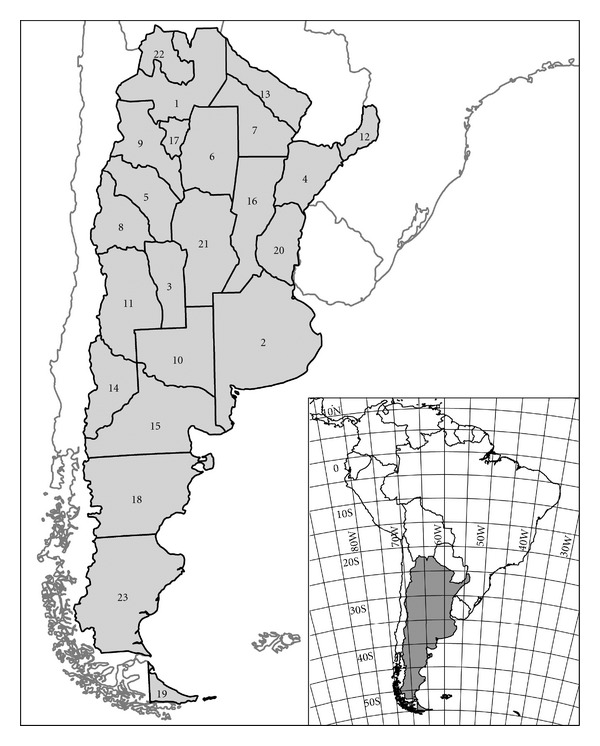
Political-administrative provinces of Argentina. 1: Salta, 2: Buenos Aires, 3: San Luis, 4: Corrientes, 5: La Rioja, 6: Santiago del Estero, 7: Chaco, 8: San Juan, 9: Catamarca, 10: La Pampa, 11: Mendoza, 12: Misiones, 13: Formosa, 14: Neuquén, 15: Río Negro, 16: Santa Fe, 17: Tucumán, 18: Chubut, 19: Tierra del Fuego, 20: Entre Ríos, 21: Córdoba, 22: Jujuy, 23: Santa Cruz.

We used the twenty-three political-administrative provinces of Argentina as territorial units (Figure 1) because it is on this kind of unit that mammal distributions are best known and, consequently, distribution data are more reliable. In addition, provinces are one of the administrative levels for the application of conservation measures; therefore, it can be useful for biological studies to perform analyses at this level [27]. Due to the artificial nature of some of their boundaries, the use of administrative provinces is sometimes criticized [24, 28], although often simultaneously recommending the use of even more artificial divisions such as latitude × longitude or UTM grid cells. 

### 2.2. The Variables

**Figure 2:**
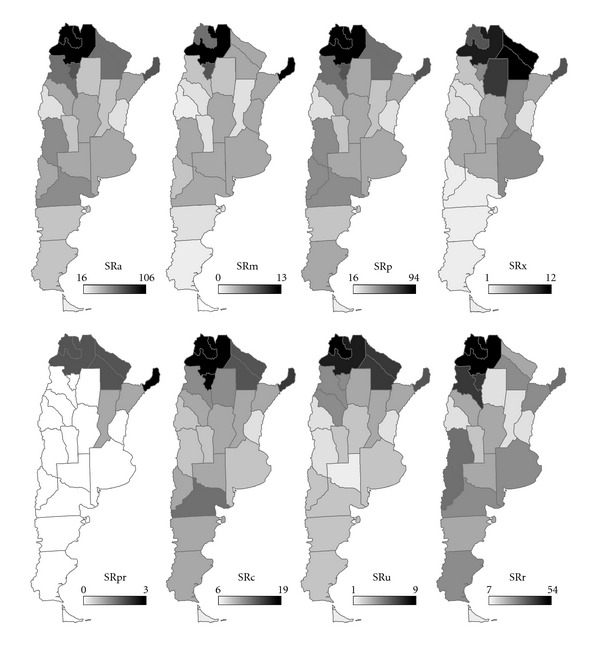
Species richness of indigenous nonvolant terrestrial mammals in each Argentinean province. SRa: all mammals; SRm: Marsupialia; SRp: Placentaria; SRx: Xenarthra; SRpr: Primates; SRc: Carnivora; SRu: Ungulates; SRr: Rodentia.

In each Argentinean province we recorded the total number of indigenous nonvolant terrestrial mammal species (SRa). Since different mammal groups can show different SR patterns both at a continental scale [29] and at a regional scale [24], we also computed the number of species for: Marsupialia (SRm), Placentaria (SRp), and, among the latter, Xenarthra (SRx), Carnivora (SRc), Ungulates (Artiodactyls + Perissodactyls, SRu), and Rodentia (SRr). Primates were not analysed as a separate group because their very low SR precluded statistical tests.  Figure 2 shows the SR obtained for each mammal group and province.

Distribution data were obtained from Cabrera and Yepes [30], Galliari et al. [31], Parera [32], Wilson and Reader [33], Barquez et al. [34], Teta et al. [35], Agnolin et al. [36], and Udrizar Sauthier et al. [37]. We used the same distribution data as Real et al. [24] but updated with more recent compilations of Argentinean species. 

From the I. G. M. [38] we obtained the values of ten environmental variables related to climate, orography, and habitat diversity, and two geographical variables: latitude and longitude (Table 1). We then performed a principal components analysis using latitude and longitude to create two new spatial variables defining the main spatial axes of Argentina (along longitude and latitude), the first of which should have an eigenvalue higher than 1, to be used as a third geographical variable representative of the main spatial gradient resulting from the combination of longitude and latitude.

In addition, we derived from latitude and longitude a short-distance connectivity matrix which was used to obtain spatial variables or filters based on spatial eigenvector mapping (SEVM) (see [39]). The eigenvectors with higher eigenvalues represent broad-scale variation and those with small eigenvalues represent fine-scale variation [39]. These spatial filters are related to the main distance axes for Argentinean provinces and reflect the spatial form of the country, irrespective of latitudinal or longitudinal orientation. We used these filters as spatial variables to assess if the distribution trends in SR were related to spatial variations other than strictly geographical (latitudinal or longitudinal) variations.

### 2.3. Geographical and Spatial Analyses

The frequency distributions of all variables were tested for normality using the Kolmogorov-Smirnov test, with the aim of eliminating from subsequent analyses those variables whose distribution was significantly (*P* < 0.001) different from normal. Then, for each mammal group, we determined which geographical variables were significantly associated with SR using Pearson's correlation analysis. A significant correlation between SR and a geographical variable was considered to reflect a geographic trend (latitudinal, longitudinal, or mixed) of that group of mammals. We identified significant spatial trends (related to the spatial form of Argentina irrespective of geographic orientation) similarly, but replacing the geographical variables with the spatial filters.

To assess if a geographic trend had an environmental explanation, we proceeded as follows.

(1) We performed Pearson's correlation analysis between each environmental variable and SR and the geographical variables included in the geographic trends. Any environmental variable significantly correlated simultaneously with SR and a geographical variable was considered as a possible explanatory variable for the geographic trend. 

(2) The set of possible explanatory variables were then used in a multiple stepwise linear regression procedure to select a subset among them and eliminate possible redundant explanatory variables. To avoid the increase in type I errors due to multiple testing [40, 41], we controlled the false discovery rate (FDR) using the procedure proposed by Benjamini and Hochberg [40], accepting only the variables that were significant under an FDR of *q* < 0.05. Residuals of the regression functions were examined and tested for autocorrelation using Moran's I spatial autocorrelation statistic [42]. We used the variance inflation factor (VIF) to quantify collinearity when more than one explanatory variable was included in the models. VIF is a positive value representing the overall correlation between each predictor and all the others in a model. VIF > 3 indicates “moderate or high” collinearity [43].

(3) We performed a Pearson's partial correlation analysis between SR and the geographical variable involved in the trend, controlling for the selected subset of possible environmental explanatory variables. If this partial correlation was not significant, then those environmental variables could be considered responsible for the geographic trend. To assess if the difference in area between the provinces could interfere with these results, we also performed Pearson's partial correlation between SR and the geographical variables controlling for the environmental variables and area simultaneously.

We also followed steps 1–3 to assess if a spatial trend was environmentally explained.

To assess if a geographical trend could be attributed to the spatial form of Argentina rather than to a geographical variable (La, Lo, or the combination of both) *per se*, we performed a Pearson's partial correlation analysis between SR and the geographical variables controlling for the spatial filters, with nonsignificant partial correlations indicating that the trend was due to the form of the country. Similarly, we assessed if a spatial trend could be attributed to a geographical variable rather than to the form of the country.

We used a variation partitioning procedure ([44], page 531) to determine whether a geographic trend was totally accounted for by the explanatory environmental variables, or if a sizable pure geographical trend (PGT) remained unaccounted for. The part of the variation in SR that follows a geographic trend was estimated using the coefficient of determination of the linear regression of SR on the geographic variable (*R*_Geogr_^2^). We then performed for each geographic trend a multiple linear regression of SR on the geographical variable and the explanatory variables of the spatial trend (*R*_*T*_^2^). The environmentally explained part of the variation in SR was estimated using the coefficient of determination of the linear regression of SR on the environmental variables included in the model (*R*_Env_^2^). The pure geographic trend was obtained by subtracting from *R*_*T*_^2^ the environmentally explained variation (*R*_p Geogr_^2^ = *R*_*T*_^2^ − *R*_Env_^2^). Then, the part of the geographic trend that was accounted for by the environmental variables was obtained by subtracting from *R*_Geogr_^2^ the pure geographic trend (*R*_EnvGeogr_^2^ = *R*_Geogr_^2^ − *R*_p Geogr_^2^). Finally, we calculated the percentage of the total geographical trend attributable to environmental causes and the percentage attributable to the pure geographic effect.

All statistical analyses were performed using IBM SPSS statistics 19 and spatial analysis in macroecology (SAM) software, version 4.0, which is freely available at http://www.ecoevol.ufg.br/sam/ [45, 46].

## 3. Results

Principal components analysis of latitude and longitude detected a main axis in Argentina that is mostly latitudinal, following the country's shape and orientation, and describes 72.4% of the geographical variation in this country (NNE-SSW axis, eigenvalue = 1.448). 

We obtained eight spatial filters (SF_1_ to SF_8_) based on SEVM extracted from the same short-distance connectivity matrix, which was truncated at a distance of 360.364 km. The first five spatial filters (SF_1_ to SF_5_) had eigenvalues higher than 1. All but SF5 were related to some of the main geographical variables (latitude, longitude and the NNE-SSW axis) (Table 2).

None of the variables considered in this study had a distribution significantly different from normal, and therefore nor did the subsequent residuals of their linear regressions [47].

### 3.1. Geographical and Spatial Trends in the Species Richness of All Mammals (SRa)

Two significant correlations were detected between the SR of all mammals (SRa) and geographical variables. Latitude was the most important, followed by the NNE-SSW axis (Table 3). Both yielded a negative correlation coefficient, which means that SRa decreases towards the south and the south-south-west.

Two environmental variables, annual precipitation range (PR) and mean altitude (MA), were also significantly correlated with SRa (Table 3), but neither of them was related to latitude (La) nor to the NNE-SSW axis (Table 3). Thus, they cannot be considered responsible for these geographical trends in the overall diversity of Argentinean mammals. 

SRa was significantly correlated with a spatial variable (SF_1_). The mean annual temperature (MT) and mean temperature of the coldest month (CT) were also significantly correlated with SF1, but neither of them was related to SRa (Tables 3 and 4), which suggests that the increase of SRa with SF_1_ cannot be related to these environmental variables.

### 3.2. Geographical and Spatial Trends in the Species Richness of Marsupials (SRm)

When mammals were divided into their two main taxonomical groups, marsupials and placentals, the results differed. For marsupials (SRm) we detected three geographical gradients: a main gradient related to the NNE-SSW axis, another one related to latitude, and a third one related to longitude (Table 3). According to these trends, SRm decreases towards the south-south-west, the south, and the west of the country, respectively. 

The environmental variables significantly correlated with SRm were CT and MP (mean annual precipitation) (Table 3). Both variables were significantly correlated with the NNE-SSW axis and to longitude, whereas CT alone was correlated with latitude (Table 4). Stepwise regression of SRm on these variables only selected CT, yielding the following equation: SRm = 0.934 + 0.359 CT, with *R* ^2^ = 0.241. The partial correlation between SRm and the NNE-SSW axis controlling for CT was significant (*r*_SRm-NNE-SSW Axis/CT_ = −0.485, *P* < 0.05), which indicates that CT is not the only cause of this geographical trend of marsupial diversity. However, the partial correlation between SRm and latitude controlling for CT was not significant (*P* > 0.05), which indicates that CT explains the latitudinal gradient of marsupial SR. The partial correlation between SRm and longitude controlling for CT was also nonsignificant, suggesting that CT is also the cause of the longitudinal trend of marsupial diversity. CT thus explains 24.1% of the variation in marsupial SR. 

There was no spatial trend of SRm along spatial filters.

### 3.3. Geographical and Spatial Trends in the Species Richness of Placentals (SRp)

For placentals (SRp), two geographical trends were detected, the most important one related to latitude and the second one related to the NNE-SSW axis (Table 3). 

The environmental variables correlated with SRp were the annual precipitation range (PR), mean altitude (MA), and altitude range (AR) (Table 3). However, their correlations with latitude were not significant (Table 4) and, therefore, cannot be considered possible causes of the latitudinal trend in placental diversity. The same situation occurred for the NNE-SSW geographical gradient.

For placentals we found two spatial trends correlated with SF_1_ and SF_3_. None of the variables significantly correlated with SF_1_ were also significantly correlated with SRp (Tables 3 and 4), and therefore the increase in SRp with SF_1_ cannot be related to the environmental variables. However, the precipitation range (PR) was significantly correlated with SF_3_ and also with SRp. This variable was selected in the stepwise regression of SRp (SRp = 30.69 + 0.031 PR, with *R* ^2^ = 0.225). Partial correlations between SRp and SF_3_ controlling for PR were not significant (*r*_SRp-SF3/PR_ = 0.232, *P* > 0.05), which suggests that the spatial gradient SF_3_ displayed by placentals is due to PR. 

Placentals were then divided into four subgroups: xenarthrans, carnivores, ungulates (which comprise Artiodactyls and Perissodactyls), and rodents. 

### 3.4. Geographical and Spatial Trends in the Species Richness of Xenarthrans (SRx)

Xenarthran SR (SRx) showed significant correlations with latitude, the NNE-SSW axis, and longitude (Table 3). Mean annual temperature (MT), mean temperature of the hottest month (HT), and mean temperature of the coldest month (CT) were significantly correlated with SRx (Table 3), and also correlated significantly with the three geographical variables (Table 3). 

Stepwise regression of SRx on these three environmental variables selected only CT (SRx = 0.579 + 0.512 CT, with *R* ^2^ = 0.50). Partial correlations between SRx and each of the geographical variables controlling for CT were not significant, which suggests that the three geographical gradients displayed by xenarthrans are due to CT. Therefore, xenarthrans are more diverse in the north, the north-north-west and the east of the country because the mean temperature of the coldest month is higher.

For SRx we found two spatial trends with SF_1_ and SF_4_. Mean annual temperature (MT) and mean temperature of the coldest month (CT) were significantly correlated with SF_1_ and with SRx (Tables 3 and 4). Stepwise regression of SRx on these two environmental variables selected only CT (SRx = 0.579 + 0.512 CT, with *R* ^2^ = 0.50). The partial correlation between SRx and SF_1_ controlling for CT was significant (*r*_SRx-SF1/CT_ = 0.469, *P* < 0.05), which indicates that CT is not the only cause of this spatial trend of xenarthran diversity.

Mean annual temperature (MT), mean temperature of the hottest month (HT), and mean temperature of the coldest month (CT) were significantly correlated with SF_4_ and SRx (Tables 3 and 4). Stepwise regression of SRx on these three environmental variables selected only CT (SRx = 0.579 + 0.5123 CT, with *R* ^2^ = 0.50). The partial correlation between SRx and SF_4_ controlling for CT was not significant (*r*_SRx-SF4/CT_ = −0.277, *P* > 0.05), which indicates that this spatial trend is likely due to CT.

### 3.5. Geographical and Spatial Trends in the Species Richness of Carnivores (SRc)

Two significant geographical trends were detected for carnivore SR (SRc): latitudinal and along the NNE-SSW axis. However, PR was the only variable that significantly correlated with SRc, and it did not correlate significantly with either geographical variable (Tables 3 and 4). Thus, no environmental explanation was found for these geographical gradients in SRc. 

We found a significant correlation between SRc and SF_1_, but the environmental variables that were significantly correlated with SF_1_ (MT and CT) were not correlated with SRc (Tables 3 and 4).

### 3.6. Geographical and Spatial Trends in the Species Richness of Ungulates (SRu)

Ungulate species richness (SRu) showed a mainly latitudinal gradient and another trend related to the NNE-SSW axis (Table 3). Two environmental variables (MT and CT) were significantly correlated with both SRu and these geographical variables (Tables 3 and 4). Stepwise regression of SRu on these environmental variables yielded the following equation: SRu = 1.390 + 0.283 CT, with *R* ^2^ = 0.327. Again, only CT was selected. The significant correlation between SRu and the NNE-SSW axis disappeared when CT was kept constant, but this did not happen with the latitudinal gradient (*r*_RSu-La/CT_ = −0.536, *P* < 0.05). Hence, CT explains the NNE-SSW trend of SRu, accounting for 32.7% of its variation.

SRu presented a significant spatial trend with SF_1_. The environmental variables that significantly correlated with SF_1 _(MT and CT) were also correlated with SRu (Table 3 and Table 4). Stepwise regression of SRu on these variables yielded the following equation: SRu = 1.390 + 0.283 CT, with *R* ^2^ = 0.327. Only CT was selected. The significant correlation between SRu and SF_1_ did not disappear when CT was kept constant (*r*_RSu-SF1/CT_ = 0.702, *P* < 0.1). Hence, CT does not explain this spatial trend of ungulate diversity.

### 3.7. Geographical and Spatial Trends in the Species Richness of Rodents (SRr)

For rodents (SRr), only the latitudinal gradient was significant (Table 3). However, the environmental variables correlated with SRr (PR, MA, and AR, Table 3) were not significantly correlated with latitude (Table 4). Therefore, we could not environmentally explain the latitudinal trend of rodent SR in Argentina.

SRr showed a significant spatial gradient with SF_3_. The annual precipitation range (PR) was significantly correlated with SF_3_ and also with SRr (Tables 3 and 4). This environmental variable was selected in the stepwise regression of SRp (SRp = 15 + 0.0203 PR, with *R* ^2^ = 0.239). Partial correlations between SRr and SF_3_ controlling for PR were not significant (*r*_RSr-SF3/PR_ = 0.349, *P* > 0.5), which suggests that the spatial gradient SF_3_ displayed by rodents is due to PR.

Results of the geographical and spatial trends for each group are summarized in Table 5.

### 3.8. Geographical versus Spatial Trends

All mammal groups showed significant geographical trends and all except marsupials presented also spatial trends (Table 3). All groups exhibited latitudinal trends, none of which seemed to be a reflection of the spatial filters. For the groups presenting spatial trends significantly correlated with latitude (SRa, SRp, and SRx) the latitudinal trend was not attributable to the spatial form of the country, because the partial correlations between SR and La, after controlling for the spatial variables (SF_1_, SF_4_), remained significant (Table 6). However, the spatial trends of these groups were mostly attributable to the effect of latitude, as the partial correlations between SR and the spatial variables (SF_1_, SF_4_) after controlling for La were not significant, except for the correlation of SRx-SF1. 

The geographical trends of SRa, SRp, SRc, SRu with the NNE-SSW axis were mainly due to the spatial form of Argentina, because the partial correlations between SR and NNE-SSW axis, after controlling for spatial variables (either SF_1_, SF_3_, or SF_4_), were not significant, whereas the partial correlation between SR and the spatial variables remained significant after controlling for the NNE-SSW axis (Table 7).

### 3.9. The Pure Geographical Trends

When we controlled for the environmental variables and area simultaneously in the partial correlation between SR and the geographical trends, the significance of all correlations was similar to that obtained when controlling only for the environmental variable, which suggests that the above mentioned results are not spurious consequences of the effect of area on SR.

Moran's I residuals for the marsupial and rodent SR models indicated statistically nonsignificant spatial autocorrelation; for xenarthrans and ungulates they indicated statistically significant spatial autocorrelation up to approximately 180 km; and for placentals they indicated statistically significant spatial autocorrelation up to approximately 346 km. We did not consider this to be a modelling problem, because the distance of spatial autocorrelation was small relative to the average size of the Argentinean provinces [42].

Table 7 shows the results of the variation partitioning of Argentinean mammal SR. For most geographical trends detected in the different mammal groups, at least 10% of the variation could be attributed to a pure geographical effect. In gradients related to latitude (La, NNE-SSW axis), the percentage of the geographical trend attributable to a pure geographic effect (% PGT) was greater than for the longitudinal gradient, especially where the pure geographical trends were significant according to the partial correlation analysis. For xenarthrans, the longitudinal trend was totally explained by climate, as the pure geographical trend was zero. 

## 4. Discussion

### 4.1. Relationships of the Latitudinal Gradient with Area and the Form of the Country

It might be thought that the latitudinal trends of SR could be related to the available area, as Argentina approximately forms an inverted isosceles triangle with higher SR in the larger area (base of the triangle in the north) than in the smaller area (tip of the triangle in the south). However, in general, the larger provinces are in the south of the country and the smallest ones are in the north (see Figure 1), and thus the cause of the lower numbers of species in the southern provinces (or the higher numbers in the northern provinces) is not the available area. In fact, the relationship between province area and the number of species was negative (although not significant), that is, larger provinces did not contain more species (Table 3). In a similar way, area has little to do with the latitudinal gradient of bats, with that of different groups of mammals in New World [18, 48] or with other taxa that exhibit strong latitudinal gradients in the Western Hemisphere [10].

On the other hand, there is the question of whether the latitudinal gradient is in fact a reflection of the spatial form of Argentina, which is predominantly aligned north to south, possibly forcing SR to vary along this spatial axis. In Argentina, one of the largest countries in the world, we detected a significant latitudinal gradient in SR of all mammals combined and in SR of every subgroup of mammals (Table 3). Spatial filters were also related to the SR of mammals and every subgroup except marsupials (Table 3). However, our results showed that the latitudinal trend is not attributable to the spatial form of the country, because the partial correlations between SR and latitude, after controlling for the spatial variables, remained significant (Table 6). Conversely, the geographical trends along NNE-SSW axis were related to the spatial form of the country.

Moreover, we found significant longitudinal gradients for marsupials and xenarthrans. These gradients were not attributable to the spatial form of the country, because for these mammal groups the number of species was not related to the spatial filters associated with longitude. Consequently, the longitudinal trends were also nonexplicable in terms of the spatial filters.

### 4.2. Relationships of the Geographical Gradient with the Environmental Variables

Historical, geographical, and environmental factors are the most likely causes of the observed geographical trends [49]. This has been shown to occur elsewhere, both at regional [20, 24] and continental scales [18, 50].

Most latitudinal diversity gradients are in fact explained by variations in environmental variables [51–53]. Particularly, the main causes of variation in the SR of most mammal groups in Argentina are environmental [24]. However, the environmental variables considered in this study could not explain the latitudinal gradients of all mammals together (SRa), nor those of placentals (SRp), carnivores (SRc), or rodents (SRr) (Tables 3 and 4). Nevertheless, the latitudinal gradients of marsupials (SRm) and xenarthrans (SRx) were explained by the mean temperature of the coldest month (CT), as were the NNE-SSW gradients of xenarthrans (SRx) and ungulates (SRu), and the spatial gradient SF_4_ of xenarthrans (SRx). This climatic variable has also been shown to explain other latitudinal gradients, for example, for mammals in North America [51] and for marsupials [54], bats [29], and carnivores [55] in South America.

The mean temperature of the coldest month is related to energy availability and, thus, to productivity. Cofré et al. [52] found that energy availability and productivity are the main determinants of small mammal patterns in Chile, and Hawkins and Porter [56] also found that another variable related to environmental energy, annual potential evapotranspiration, was the strongest predictor of mammal diversity in North America. Hawkins et al. [57] pointed out that the main constraint on animal richness in the Southern hemisphere is water rather than energy, with the exceptions of Argentinean reptiles and birds. At a more regional scale, Diniz-Filho et al. [58] found that the simultaneous availability of water and energy (annual actual evapotranspiration) was the most important predictor of mammal and bird diversity in the Brazilian Cerrado. However, our results suggest that, for the latitudinal gradient of Argentinean mammal diversity, energy is more important than water availability.

The other gradient detected in this study, namely, the longitudinal trend of marsupial and xenarthran SR, is not as common or general as the latitudinal trend. We also found that these gradients were related to the mean temperature of the coldest month (CT). Rodriguero and Gorla [59] found a longitudinal gradient for Triatominae (Insecta: Hemiptera: Heteroptera) SR in South America related to temperature. The higher SR values occurred at eastern longitudes, where the highest temperatures occur, decreasing then progressively towards the Andes. The species-energy hypothesis can account for this longitudinal gradient in SR.

The annual precipitation range (PR) was related to the spatial gradients SF_3_ of placentals (SRp) and rodents (SRr) and represents climatic heterogeneity within the provinces, as it describes the difference in annual precipitation between the wettest and the driest parts of the province.

Given that climate explains a sizable part of the geographical variations of mammal diversity in Argentina, it would be interesting to consider historical climatic variables to explain past distributions [60, 61], and to apply climatic change variables, as suggested by Dunn et al. [62], to forecast future effects of climate on mammal species diversity. Moreover, the study of biotic interactions [13] might provide additional understanding of the mechanisms that generate latitudinal gradients.

### 4.3. The Pure Geographical Trends

Most of the geographical trends were not explained by the environmental variables, so they might be related to purely historical, purely geographical, or other factors. Even in the explained geographical gradients, we showed that there was a purely geographical effect that was only related to latitude (see Table 7). Therefore, latitude may not be as spurious for mammals as it is sometimes believed to be [19–22]. Latitude not only affects temperature patterns and related environmental conditions (which it does because of its relationship with the position of the sun), but also produces the so-called Coriolis effect, which affects air and water masses moving along latitudinal paths, affecting ocean and atmospheric currents with implications on the distribution patterns of many marine and terrestrial organisms. Latitude also affects the geographical-topological relationships between different locations (e.g., two points separated by the same longitude are closer at higher latitudes, facilitating the convergence of migrant populations at these latitudes) and roughly corresponds to the geomagnetic field which may be felt by many species.

## Figures and Tables

**Table 1:** Environmental, geographical, and spatial variables used for analysing the geographical gradients in terrestrial mammal species richness in the Argentinean provinces. SEVM: Spatial eigenvector mapping.

Environmental variables	
MT: Mean annual temperature	
HT: Mean temperature of the hottest month	
CT: Mean temperature of the coldest month	
TR: Annual temperature range	
MP: Mean annual precipitation	
PR: Annual precipitation range	
MA: Mean altitude	
AR: Altitude range	
LR: Latitude range	
SA: Surface area	

Geographical variables	
La: Mean latitude	
Lo: Mean longitude	
NNE-SSW: mean value in the NNE-SSW axis	

Spatial variables	
SF_*i*_: Spatial Filters (SEVM)	

(Source: [38]).

**Table 2:** Pearson's correlation coefficients between the spatial filters and the geographical variables.

	SF_1_	SF_2_	SF_3_	SF_4_	SF_5_
La	−0.576**	−0.239	0.219	0.663**	0.082
Lo	−0.345	0.572**	0.516*	0.326	−0.012
NNE-SSW axis	−0.541**	0.195	0.432*	0.581**	0.041

Statistically significant correlations are in bold. *P < 0.05, **P < 0.01. Variable codes as in Table 1.

**Table 3:** Pearson's correlation coefficients between the species richness of each group of mammals and the geographical, spatial, and environmental variables used for analysing the geographical gradients in species richness.

	Variables	SRa	SRm	SRp	SRx	SRc	SRu	SRr
Geographical	La	−0.647**	−0.566**	−0.638**	−0.733**	−0.670**	−0.721**	−0.437*
	Lo	−0.159	−0.480*	−0.099	−0.539**	−0.187	−0.297	0.143
	NNE-SSW axis	−0.474*	−0.614**	−0.433*	−0.748**	−0.504*	−0.599**	−0.172

Spatial	SF_1_	0.578**	0.389	0.590**	0.660**	0.672**	0.793**	0.358
	SF_3_	0.394	0.162	0.420*	−0.010	0.333	0.178	0.514*
	SF_4_	−0.340	−0.391	−0.320	−0.556**	−0.334	−0.305	−0.170

Environmental	MT	0.32	0.331	0.307	0.637**	0.328	0.474*	0.104
	HT	0.17	0.215	0.156	0.423*	0.075	0.201	0.073
	CT	0.368	0.491*	0.334	0.707**	0.396	0.572**	0.07
	TR	−0.131	−0.192	−0.116	−0.152	−0.269	−0.287	0.032
	MP	0.136	0.476*	0.073	0.23	0.185	0.168	−0.081
	PR	0.464*	0.311	0.474*	0.148	0.494*	0.288	0.489*
	MA	0.427*	0.141	0.461*	−0.144	0.364	0.238	0.625**
	AR	0.407	0.138	0.438*	−0.156	0.339	0.213	0.604**
	LR	−0.204	−0.291	−0.182	−0.039	−0.331	−0.212	−0.099
	SA	−0.119	−0.172	−0.105	−0.168	−0.252	−0.256	0.044

Statistically significant correlations are in bold (*P < 0.05, **P < 0.01). Variable codes as in Table 1 and Figure 2.

**Table 4:** Pearson's coefficients measuring the correlation of the environmental variables with the geographical and spatial variables.

	MT	HT	CT	TR	MP	PR	MA	AR	LR	SA
La	−0.865**	−0.606**	−0.797**	0.022	−0.169	0.040	−0.180	−0.137	0.137	0.369
Lo	−0.554**	−0.473*	−0.779**	0.169	−0.802**	0.172	0.631**	0.625**	0.049	0.181
NNE-SSW axis	−0.833**	−0.634**	−0.926**	0.112	−0.571**	0.124	0.265	0.287	0.110	0.323
SF_1_	0.456*	0.134	0.537**	−0.334	0.179	0.220	0.035	0.023	−0.287	−0.307
SF_3_	−0.432*	−0.351	−0.441*	0.002	−0.316	0.517*	0.325	0.312	−0.204	0.144
SF_4_	−0.636**	−0.495*	−0.557**	−0.066	−0.199	0.070	0.093	0.127	0.047	0.188

Statistically significant correlations are in bold (*P < 0.05, **P < 0.01). Variable codes as in Table 1 and Figure 2.

**Table:** (a)

Group	Geographical trend	Pearson's correlation			Pearson's partial correlation
		*r* SR-Env	*r* Geog-Env	Stepwise regression	*r* SR-Geog/Env
SRa	La	PR	—		
		MA	—		
	NNE_SSW axis	PR	—		
		MA	—		

SRm	La	**CT**	**CT**	**CT**	**n.s**
		M			
	Lo	**CT**	**CT**	**CT**	**n.s.**
		MP	MP		
	NNE_SSW axis	CT	CT	CT	*
		MP	MP		

SRp		PR	—		
	La	MA	—		
		AR	—		
		PR	—		
	NNE_SSW axis	MA	—		
		AR	—		

SRx		MT	MT		
	La	HT	HT		
		**CT**	**CT**	**CT**	**n.s.**
		MT	MT		
	Lo	HT	HT		
		**CT**	**CT**	**CT**	**n.s.**
		MT	MT		
	NNE_SSW axis	HT	HT		
		**CT**	**CT**	**CT**	**n.s.**

SRc	La	PR	—		
	NNE_SSW axis	PR	—		

SRu	La	MT	MT		
		CT	CT	CT	*
	NNE_SSW axis	MT	MT		
		**CT**	**CT**	**CT**	**n.s**

SRr		PR	—		
	La	MA	—		
		AR	—		

**Table:** (b)

		Pearson's correlation			Pearson's partial correlation
Group	Spatial trend	*r* SR/Env	*r* SF/Env	Stepwise regression	*r* SR-Geog/Env
SRa	SF_1_	PR	—		
		MA	—		

SRp		PR	—		
	SF_1_	MA	—		
		AR	—		
		**PR**	**PR**	**PR**	**n.s.**
	SF_3_	MA	—		
		AR	—		

SRx		MT	MT		
	SF_1_	HT			
		CT	CT	CT	*
		MT	MT		
	SF_4_	HT	HT		
		**CT**	**CT**	**CT**	**n.s.**

SRc	SF_1_	PR	—		

SRu	SF_1_	MT	MT		
		CT	CT	CT	*

SRr		**PR**	**PR**	**PR**	**n.s.**
	SF_3_	MA			
		AR			

**Table 6:** A: Partial correlation coefficients between SR and the geographical trends (Latitude and NNE-SSW axis) controlling for spatial variables (SF_1_, SF_3_, SF_4_). B: Partial correlation coefficients between SR and the spatial variables (SF_1_, SF_3_, SF_4_) controlling for the geographical variables (Latitude and NNE-SSW axis). Nonsignificant (n.s.) coefficients indicate that the trend displayed by SR is due to the controlled variable (*P < 0.05, **P < 0.01).

A	B
*r*_SRa-La/SF1_ = −0.471*	*r*_SRa-La/SF1_ = 0.329 n.s.
*r*_SRa-La/SF1_ = −0.452*	*r*_SRp-SF1/La_ = 0.353 n.s.
*r*_SRx-La/SF1_ = −0.575**	*r*_SRx-SF1/La_ = 0.427*
*r*_SRx-La/SF4_ = −00586**	*r*_SRx-SF4/La_ = −0.139 n.s.
*r*_SRc-La/SF1_ = −0.467*	*r*_SRc-SF1/La_ = 0.472*
*r*_SRu-La/SF1_ = −0.532*	*r*_SRu-SF1/La_ = 0.667**
*r*_SRa-Axis1/SF1_ = −0.235 n.s.	*r*_SRa-SF1/Axis1_ = 0.434*
*r*_SRp-Axis1/SF1_ = −0.168 n.s.	*r*_SRp-SF1/Axis1_ = 0.469*
*r*_SRp-Axis1/SF3_ = −0.751**	*r*_SRp-SF3/Axis1_ = 0.747**
*r*_SRx-Axis1/SF1_ = −0.618**	*r*_SRx-SF1/Axis1_ = 0.457*
*r*_SRx-Axis1/SF4_ = −0.627**	*r*_SRx-SF4/Axis1_ = −0.226 n.s.
*r*_SRc-Axis1/SF1_ = −0.225 n.s.	*r*_SRc-SF1/Axis1_ = 0.550**
*r*_SRu-Axis1/SF1_ = −0.331 n.s.	*r*_SRu-SF1/Axis1_ = 0.696**

**Table 7:** Variation partitioning of species richness (SR). The shown values are the proportion of variation in SR corresponding to a geographical trend attributable to environmental causes (EGT), the proportion corresponding to a pure geographic trend (PGT), the percentage of the total geographical trend environmentally explained (% EGT) and the percentage of the geographic trend attributable to a pure geographic effect (% PGT). Variable codes as in Table 1 and Figure 2. In bold are the values corresponding to pure geographic trends that are significant according to the partial correlation analysis.

Mammal group	Geographical Gradient	EGT	PGT	%EGT	%PGT
SRm	La	0.237	0.083	74,06	25.94
SRx		0.458	0.079	85,29	14.714
SRu		0.327	**0.194**	62,76	**37.24**

SRm	NNE-SSW axis	0.198	**0.179**	52,52	**47.484**
SRx		0.499	0.06	89,27	10.734
SRu		0.324	0.034	90,50	9.50

SRm	Lo	0.206	0.024	89,56	10.43
SRx		0.291	0.00	100	0.00
